# Estudo Morfofuncional do Átrio Esquerdo Isolado de um Modelo Experimental de Hipertensão Pulmonar em Ratos

**DOI:** 10.36660/abc.20230188

**Published:** 2023-10-02

**Authors:** Jorge Lucas Teixeira-Fonseca, Julliane Vasconcelos Joviano-Santos, Fabiana da Silva Alcântara, Polyana Leal da Silva, Michael Ramon Lima Conceição, Danilo Roman-Campos

**Affiliations:** 1 Universidade Federal de São Paulo São Paulo SP Brasil Universidade Federal de São Paulo , São Paulo , SP – Brasil; 2 Faculdade de Ciências Médicas de Minas Gerais Belo Horizonte MG Brasil Faculdade de Ciências Médicas de Minas Gerais , Belo Horizonte , MG – Brasil

**Keywords:** Átrios do Coração, Fibrose, Hipertensão Pulmonar, Arritmias Cardíacas, Monocrotalina

## Abstract

**Fundamento:**

A alta incidência de arritmias atriais na hipertensão pulmonar (HP) pode estar associada a um prognóstico ruim, e o átrio esquerdo (AE) pode desempenhar um papel neste quadro. Um achado importante nos estudos de HP é que a remodelação do AE é subestimada.

**Objetivo:**

Este estudo investigou a morfologia e a função mecânica do AE, bem como a suscetibilidade ao desenvolvimento de arritmias em um modelo de HP induzida por monocrotalina (HP-MCT).

**Métodos:**

Ratos Wistar com 4 semanas de idade receberam 50 mg/kg de MCT. Foram realizadas análises eletrocardiográficas e histológicas para avaliar o estabelecimento do modelo de HP-MCT. O tecido foi montado em banho de órgão isolado para caracterizar a função mecânica do AE.

**Resultados:**

Em comparação com o grupo controle, o modelo de HP-MCT apresentou hipertrofia do AE e alterações da atividade elétrica cardíaca, conforme evidenciadas pelo aumento da duração da onda P, PR e intervalo QT. Não foi observada alteração no inotropismo do AE isolado de ratos com HP-MCT; no entanto, o tempo para atingir a contração máxima foi atrasado. Finalmente, não observamos diferença na suscetibilidade à arritmia no AE dos ratos com HP-MCT após o protocolo de estimulação intermitente.

**Conclusão:**

A remodelação morfofuncional do AE não levou ao aumento da suscetibilidade à arritmia *ex vivo* após a aplicação do protocolo de estimulação intermitente.

## Figura Central

**Figure:**
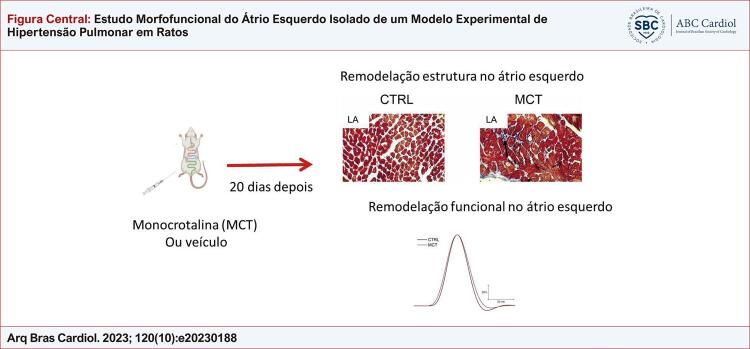
: Estudo Morfofuncional do Átrio Esquerdo Isolado de um Modelo Experimental de Hipertensão Pulmonar em Ratos

Remodelação estrutural e funcional do átrio esquerdo induzida por monocrotalina em ratos.

## Introdução

Hipertensão pulmonar (HP) é um termo genérico que se refere a um aumento na pressão média da artéria pulmonar (PMAP) ≥ 20 mmHg em repouso, avaliada por cateterização cardíaca direita. ^1^ Lesões nas pequenas artérias pulmonares, incluindo, entre outras, proliferação anormal das células musculares lisas e endoteliais na vasculatura pulmonar, ^2^ consequentemente, promove o espessamento progressivo da parede da artéria pulmonar, resultando no aumento da PMAP e gradativamente uma sobrecarga de pressão nas câmaras cardíacas. ^1^

Uma característica primária da HP é o envolvimento do lado direito do coração. Contudo, a medida que a doença evolui, o lado esquerdo do coração, incluindo o átrio esquerdo (AE), ^3^ também é afetado. ^4 , 5^ As alterações no AE são complexas e multifatoriais, incluindo aumento na área, contratilidade prejudicada, função sistólica e diastólica comprometidas. ^6 - 8^

O estiramento crônico do AE é parcialmente responsável pelas arritmias supraventriculares presentes em pacientes com HP. ^3 , 8 , 9^ Possivelmente, o teto do AE, caracterizado pela bifurcação das artérias pulmonares e do brônquio esquerdo, ^10^ seja o principal responsável pela gênese das arritmias. ^11^ Vários estudos sugerem que a assinatura estrutural, molecular e funcional do átrio direito (AD) e do AE pode ser diferente na HP. ^5 , 12 - 14^

Arritmias supraventriculares, como observadas na HP, geralmente estão associadas à gravidade da doença e estão relacionadas a um prognóstico ruim. ^15^ Assim, compreender a remodelação do AE e sua suscetibilidade ao desenvolvimento de arritmias é de extrema relevância. Existem diferentes modelos pré-clínicos para estudo de cardiopatias secundárias à HP; entre estes, o modelo animal da MCT é, sem dúvida, o mais utilizado. A MCT é um alcalóide pirrolizidínico extraído dos caules, folhas e sementes da *Crotalaria spectabilis* . Em ratos, a MCT é biotransformada no fígado em seu metabólito ativo. As lesões promovidas pela MCT são heterogêneas, incluindo lesão endotelial, espessamento medial de grandes artérias e hipertrofia cardíaca. ^4 , 5 , 16 - 21^

Um relato anterior ^5^ descreveu o comprometimento da função elétrica do AE no modelo de HP-MCT; entretanto, não se sabe se o HP-MCT prejudica a função mecânica do AE e/ou aumenta a suscetibilidade a arritmias. Assim, este estudo investiga a função mecânica do AE, 20 dias após a administração de MCT em animais controle (CTRL) e administrados com MCT.

## Métodos

### Animais

Todos os procedimentos de manejo dos animais foram aprovados pelo Comitê de Ética no Uso de Animais da Universidade Federal de São Paulo (número 9073161118). Os ratos foram obtidos no Centro para o Desenvolvimento de Modelos Experimentais para Biologia e Medicina e alojados em instalações institucionais em um ciclo claro/escuro de 12 horas com comida e água disponíveis *ad libitum* .

### Desenho Experimental para Hipertensão Pulmonar Induzida por Monocrotalina

Um método de sorteio simples foi utilizado para dividir aleatoriamente ratos Wistar machos com quatro semanas de idade e peso aproximado de 100 g em dois grupos: grupo controle (CTRL) e o grupo experimental (MCT). Após o sorteio, os animais foram reagrupados e identificados em seus respectivos grupos: 1) CTRL, que recebeu uma única dose i.p. de solução veículo de dimetilsulfóxido (DMSO, Synth®) (1 ml/kg); e 2) Grupo MCT, que recebeu uma única injeção i.p. de MCT (50 mg/kg - SIGMA Chemical Co., St. Louis, MO, EUA) dissolvido em DMSO no dia 0, conforme descrito anteriormente. ^22 , 23^ Os animais foram observados por 20 dias após a administração de MCT e DMSO.

### Medições Eletrocardiográficas In Vivo

Os ratos foram anestesiados por inalação de isoflurano 1,5–2,0% (Isoforine®, Cristália, SP, Brasil) e colocados em posição supina para a realização dos experimentos de eletrocardiograma (ECG). O registro do ECG de cinco minutos foi realizado utilizando equipamento eletrocardiográfico (ECG-PC versão 2.07 ®-TEB, MG, Brasil) antes da administração da MCT ou veículo e após 20 dias de tratamento. Os parâmetros avaliados foram duração da onda P, duração do complexo QRS, intervalos PR, intervalo QT e frequência cardíaca. Os valores do intervalo QT foram corrigidos pela fórmula de Bazett (QTc = QT/√RR). Todos os traçados de ECG foram analisados offline. Após o cálculo da média de, pelo menos, dez ciclos elétricos sucessivos para cada animal, os parâmetros foram analisados.

### Parâmetros Morfológicos

O peso corporal dos ratos dos grupos CTRL e MCT foi medido no dia 0 e no dia 20. Ao final do estudo, os ratos foram decapitados para coleta do coração. Utilizamos o índice de Fulton para expressar o grau de hipertrofia do ventrículo direito (VD), calculado a partir da razão do peso do VD pela soma do ventrículo esquerdo (VE) e septo (S), conforme descrito anteriormente. ^23 , 24^ Além disso, a relação entre o peso do coração e o comprimento da tíbia foi avaliada para determinação da hipertrofia cardíaca geral.

### Estudos Histológicos

O processamento histológico dos corações foi realizado conforme descrito anteriormente. ^25^ Os tecidos foram fixados por imersão em paraformaldeído a 4% por 24 horas a uma temperatura de 4 °C. Posteriormente, o tecido cardíaco foi desidratado com uma série crescente de etanol (70, 80, 90 e 100%), seguido de clarificação em xilol e inclusão em parafina. Em seguida, cortes transversais de 8 µm de espessura foram feitos utilizando um micrótomo (modelo HM335E; Microm, Inc., Minneapolis, MN, EUA). Após desparafinização com xileno e reidratação por meio de concentrações graduadas de etanol (100, 90, 80 e 70%), as lâminas foram coradas com Tricromo de Masson para avaliação da morfologia cardíaca, bem como da extensão da fibrose. A análise morfométrica da classificação foi realizada utilizando o software ImageJ ®, versão 1.44 (National Institutes of Health, Bethesda, MD, EUA). Nesta análise, utilizamos uma grade predefinida e uma ferramenta de contagem de células para medição, configurada com 1.000 pontos de intersecção por rato, a partir de 20 quadros, conforme descrito anteriormente. ^25^

### Preparação do átrio esquerdo

O AE foi cortado perpendicularmente e montado em cuba para órgãos isolados, contendo solução de Tyrode (mM): 140 NaCl, 5,4 KCl, 1,8 CaCl _2_ , 0,5 MgCl _2_ , 0,33 NaH _2_ PO _4_ , 11 Dextrose e 5 HEPES continuamente gaseificados com 100% de O _2_ , como descrito anteriormente. ^22 , 23 , 26^ As extremidades do AE foram suspensas horizontalmente por ganchos de aço inoxidável e equilibradas sob uma tensão de repouso de 0,5 gf (4,9 mN) por, no mínimo, 40 minutos antes do registro. O AE foi estimulado a 1 Hz.

### Protocolos de ritmo

A susceptibilidade do AE ao desenvolvimento de arritmia foi testada aplicando-se um único trem de 50 pulsos na frequência de 30 Hz através de um par de eletrodos de prata (Ag/AgCl) posicionados a 90º e conectados a um estimulador (Myopacer, IonOptix), conforme descrito anteriormente. ^27^

### Análise Estatística

Todos os dados são apresentados como média ± desvio padrão (DP). A normalidade da variável dependente foi testada com o teste de Shapiro-Wilk. As comparações entre os grupos foram realizadas usando um teste t de Student não pareado bicaudal. As variáveis contínuas foram analisadas por meio do teste t de Student pareado. O teste exato de Fisher foi utilizado para os dados categóricos (incidência de arritmias). A legenda das figuras identificam o teste estatístico utilizado. O nível de significância para rejeição de hipótese nula foi p < 0,05. A quantidade de animais, indicada na legenda da figura, é representada por (n). Os dados foram analisados utilizando Excel® (Microsoft, EUA) e Origin 8.0® (OriginLab, EUA).

## Resultados

### Análise Morfofuncional Cardíaca em Modelo Experimental de HP-MCT em Ratos

**Figura 1:**
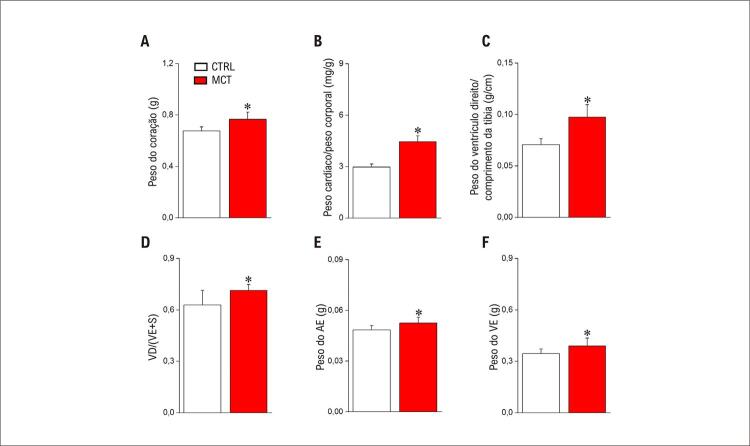
– Remodelação morfológica do coração após administração de MCT. (A) Peso cardíaco; (B) Razão entre peso cardíaco normalizado e peso corporal; (C) Peso do ventrículo direito (VD) normalizado ao comprimento do osso tibial; (D) Índice de Fulton; (E) Peso do átrio esquerdo (AE); (F) Peso do ventrículo esquerdo (VE) (n=7 animais por grupo). Os dados são expressos como média ± DP. As diferenças entre os grupos foram analisadas por meio do teste t de Student. *comparando MCT e CTRL (p < 0,05).

**Figura 2:**
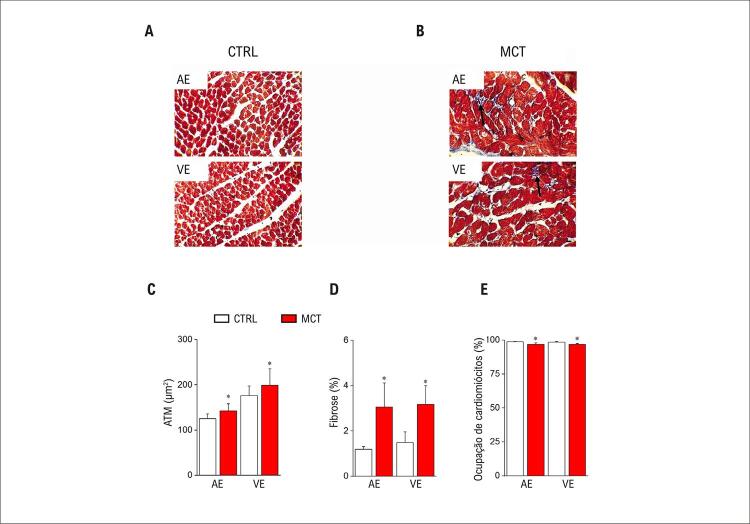
– Remodelação estrutural do coração após administração de MCT. Imagens representativas de cortes transversais de cardiomiócitos corados com Tricromo de Masson (barra de escala = 500 µm) do átrio esquerdo (AE) e ventrículo esquerdo (VE) dos animais controle (CTRL, A) e tratados (MCT, B); (C) Análise da área transversal média (ATM) dos cardiomiócitos do átrio e ventrículo esquerdos; (D) Quantificação da deposição de colágeno. As áreas fibróticas intersticiais estão coradas em azul; (E) Ocupação de cardiomiócitos (n=3-4 animais por grupo). Os dados são expressos como média ± DP. As diferenças entre os grupos foram analisadas por meio do teste t de Student. *comparando MCT e CTRL (p<0,05).

Primeiramente, foi realizada uma análise morfofuncional cardíaca para observar marcadores clássicos de doença cardíaca no modelo de ratos com HP-MCT ( Figura 1 ). Inicialmente, avaliamos o peso do coração (Figura 1A), o peso do coração normalizado pelo peso corporal (Figura 1B), o peso do ventrículo direito (VD) normalizado pelo comprimento da tíbia (Figura 1C) e o índice de Fulton (Figura 1D). Todos esses parâmetros estavam aumentados no grupo MCT em comparação com o grupo CTRL, indicando remodelação do lado direito do coração, como já demonstrado em estudos anteriores. ^5 , 22 , 23 , 28^ Além disso, observamos que os pesos do AE (Figura 1E) e do VE (Figura 1F) também estavam aumentados no grupo MCT em comparação com o grupo CTRL, sugerindo remodelamento estrutural do lado esquerdo do coração. Para caracterizar melhor o modelo de MCT, realizamos uma avaliação histológica do lado esquerdo do coração ( Figura 2 ).

Por meio da avaliação histológica, observamos que a área transversal dos miócitos do AE e do VE no grupo experimental foi aumentada em comparação ao grupo CTRL (Figuras 2A-B) e quantificada na (Figura 2C). Curiosamente, o aumento da área transversal foi observado em um cenário de aumento do conteúdo de colágeno no AE e no VE (Figura 2D) e redução da ocupação total de miócitos (Figura 2E).

**Figura 3:**
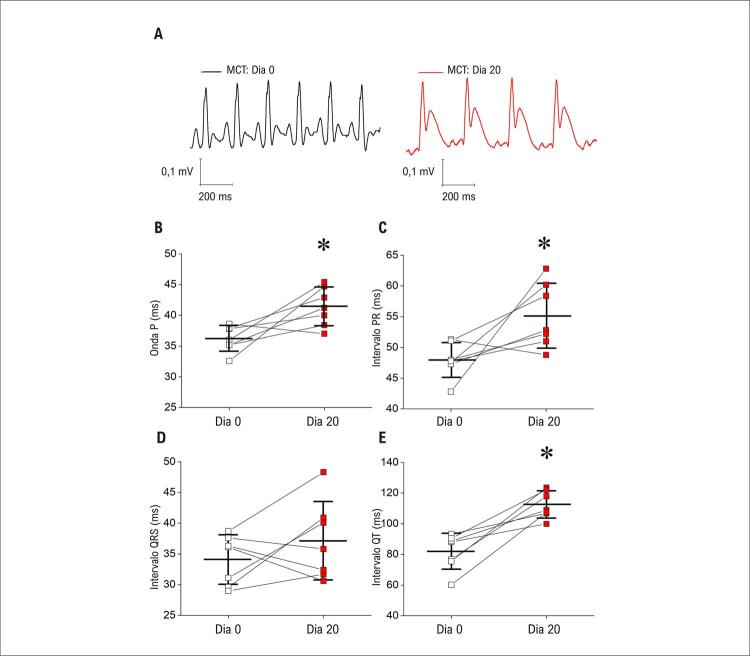
– Os parâmetros eletrocardiográficos são alterados após a administração de MCT. A) Traços representativos; B) duração da onda P; C) intervalo PR; D) duração do complexo QRS; E) Duração do intervalo QT. Os dados são expressos como média ± DP (n=7 animais por grupo). As diferenças entre os grupos foram analisadas por meio do teste t de Student pareado. *em comparação com o CTRL (p<0,05).

Assim, o modelo de HP-MCT recapitula a maior parte do fenótipo morfológico do coração observado em outros estudos. ^5 , 22 , 23 , 28^ Para confirmar esse resultado, verificamos se o modelo de HP-MCT em nosso estudo apresenta as mesmas alterações esperadas no eletrocardiograma, conforme relatadas anteriormente neste modelo animal. ^5 , 22 , 23 , 28^ O eletrocardiograma de superfície foi realizado nos grupos CTRL e MCT, e está resumido na ( Figura 3 ). Traços representativos de ECG são mostrados na (Figura 3A). Após 20 dias da administração de MCT, a duração da onda P (Figura 3B), o intervalo PR (Figura 3C) e o intervalo QT (Figura 3E) aumentaram. Não foram observadas alterações no intervalo QRS (Figura 3D). Assim, o ECG associado à análise morfométrica e histológica do coração demonstra que o modelo de rato com HP-MCT desenvolve um fenótipo cardíaco associado a estrutura e função prejudicadas, conforme descrito anteriormente. ^5 , 22 , 23 , 28^

### Função do AE em ratos dos grupos CTRL e MCT

**Figura 4:**
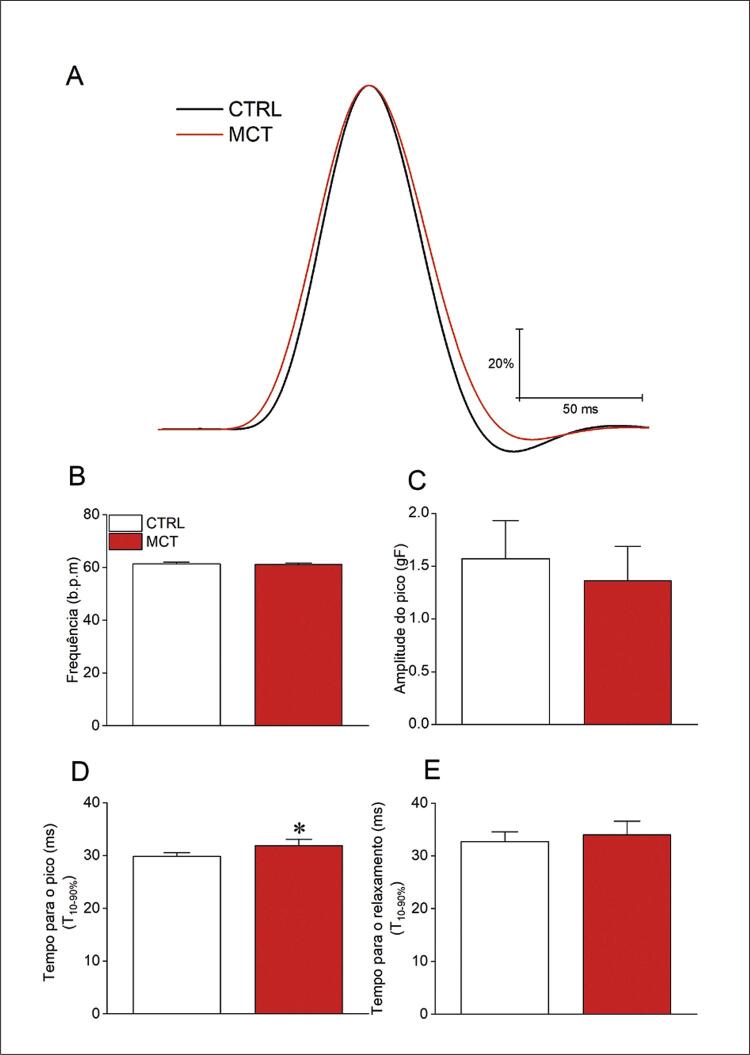
– A MCT prejudica a função do AE. A) Traçados representativos do átrio esquerdo sobreposto normalizado estimulado a 1 Hz para CTRL (n=10) e MCT (n=10); B) Frequência do átrio esquerdo; C) Amplitude máxima de contração; D) Tempo até atingir a máxima elevação de entre 10-90%; E) Tempo até atingir a máxima queda da contração entre 10-90%. Os dados são expressos como média ± DP. As diferenças entre os grupos foram analisadas por meio do teste t de Student. *em comparação com o CTRL (p<0,05).

Em seguida, conduzimos uma série de experimentos para estudar a função do AE *ex vivo* estimulado a 1 Hz. A Figura 4 resume nossos achados. Uma curva normalizada média sobreposta é mostrada na (Figura 4A) para os grupos experimentais CTRL e MCT. Não houve diferença no AE do modelo de HP-MCT quanto à frequência de estimulação (Figura 4B) e a amplitude máxima (Figura 4C). Uma diferença modesta, mas significativa, foi observada no tempo até a contração máxima (Figura 4D), medido como o tempo entre 10 e 90% para o pico da contração. Não foi observada alteração no tempo de relaxamento (Figura 4E).

### Suscetibilidade à arritmia do AE isolado de ratos do grupo MCT

**Figura 5:**
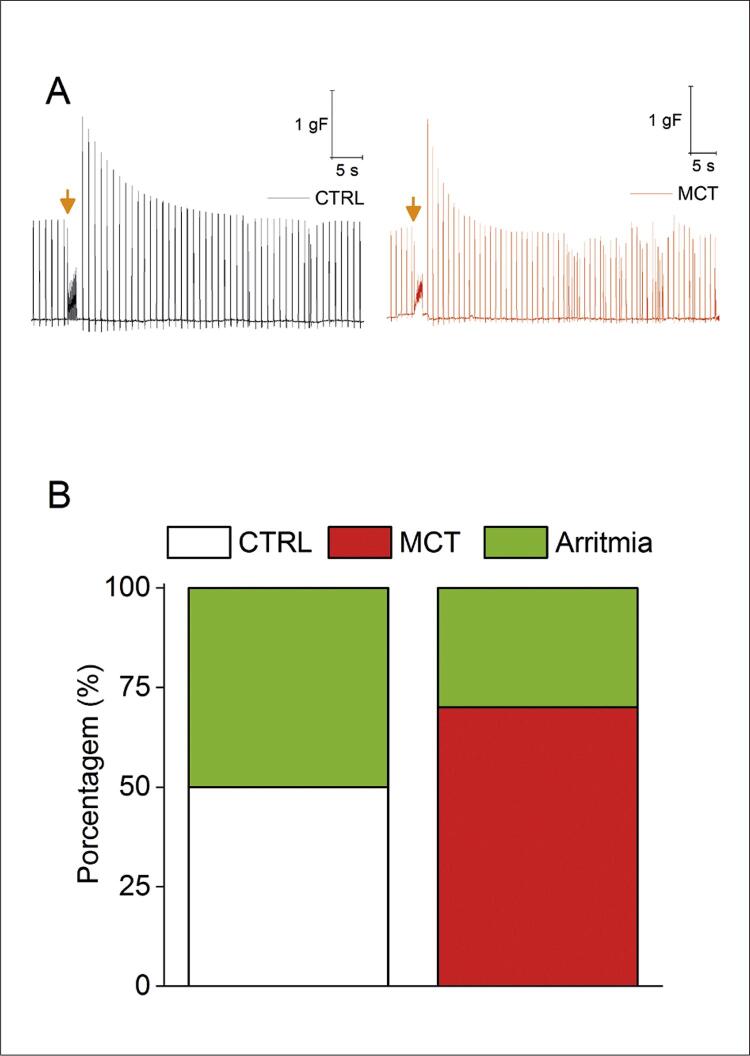
– Suscetibilidade à arritmia do AE isolado. A) Traçados representativos do AE isolado na situação CTRL (painel esquerdo) e MCT (painel direito) após o protocolo de estimulação intermitente (burst). (B) Incidência dos eventos arrítmicos. Teste Chi-quadrado, p>0,05. CTRL (n = 10) e MCT (n=10).

Um relato anterior descobriu que o AD isolado de animais com HP-MCT, após 14 dias de administração de MCT, é suscetível ao desenvolvimento de arritmias após protocolo de estimulação *ex vivo* . ^22 , 23^ Aqui, exploramos a suscetibilidade do AE de animais com HP-MCT para o desenvolvimento de arritmias após 20 dias da administração de MCT. Nossos achados foram resumidos na ( Figura 5 ). Após o protocolo de estimulação intermitente ( *burst* ), o AE do grupo CTRL desenvolveu algumas extrassístoles, conforme evidenciadas no traço representativo (Figura 5A), traço esquerdo, e o AE do grupo HP-MCT, representado na (Figura 5A), traço direito. Conforme mostrado na (Figura 5B), eventos arrítmicos foram observados na mesma frequência no grupo MCT (3/10) em comparação com o grupo CTRL (5/10) após estimulação intermitente ( *burst* ). Além disso, a maioria dos eventos arrítmicos observados foram extrassístoles e foi detectada fibrilação não atrial.

## Discussão

O modelo de HP-MCT em ratos provoca alterações complexas em nível celular e molecular, contribuindo para a remodelação do miocárdio e levando ao aumento da pressão nas cavidades cardíacas e ao desenvolvimento de hipertrofia. Em nosso estudo, o modelo HP-MCT foi estabelecido pelo aumento do peso médio do AE, do VE, do índice de Fulton e da área transversal dos cardiomiócitos. ^22 , 23^ Além disso, os resultados histológicos confirmaram a presença de fibrose tecidual; esse fenômeno reflete uma maior heterogeneidade elétrica, comprometendo a estrutura atrial e, consequentemente, aumentando a suscetibilidade a arritmias. ^28^

No presente estudo, exploramos as alterações morfofuncionais do AE após a administração de MCT em ratos. No entanto, observamos que o modelo de HP-MCT não aumentou a suscetibilidade do AE para o desenvolvimento de arritmia *ex vivo* após a aplicação do protocolo de estimulação intermitente ( *burst* ). Nossos resultados divergem do relato anterior, que utilizou um modelo experimental semelhante ao aplicado neste estudo, no qual os autores constataram que o AE ficou suscetível a desenvolver arritmias após administração *in situ* de protocolo intermitente ( *burst* ). ^5^ Eles identificaram que a suscetibilidade à arritmia estava aumentada no AE em um quadro de aumento da fibrose no tecido e aumento da duração da onda P, de maneira semelhante à observada em nossa investigação atual. ^5^

Existem algumas explicações para a aparente discrepância do nosso estudo e do relato de Hiram et al. Por exemplo, os autores aplicaram o medicamento Blebbistatina em seu preparo, que desacopla a função elétrica e mecânica do miócito. Assim, o conceito experimental estudo deles difere do nosso, uma vez que exploramos a função mecânica para avaliar a suscetibilidade à arritmia. Além disso, os autores também aplicaram um protocolo distinto para induzir arritmia, mais severo. Os autores usaram estimulação intermitente ( *burst* ) de 50 Hz, aplicada por 3 s, com 12 disparos separados por intervalos de 2 s; enquanto isso, no presente trabalho foi aplicado um único estímulo de 30 Hz.

Outra possível explicação para a diferença entre nossos resultados e os da literatura é a idade dos ratos utilizados nos estudos, que será discutida mais adiante. Além disso, podemos dizer que o tempo de evolução da HP-MCT do nosso estudo difere de outros, principalmente em relação à dose de MCT e ao peso/idade dos animais utilizados. ^5 , 22 , 23 , 28^ Aqui, relatamos o fenótipo do AE de animais pesando 100 g no início do estudo que foram tratados com 50 mg/kg de MCT, muito mais jovens que os utilizados no estudo de Hiram et al.

Sabe-se que animais com ~100 g são mais suscetíveis à administração de MCT, com mortalidade de 100% até 23 dias após o tratamento com MCT. ^18 , 29 , 30^ Uma observação interessante é que, em outro relato, notou-se que, em ratos Wistar machos, com peso de 150 a 175 g, administrados com 40 ou 60 mg/kg de MCT, após 14 dias, ambos os grupos desenvolveram pressão ventricular direita elevada, sendo mais grave no grupo que recebeu a dose de 60 mg/kg. ^31^ Além disso, ratos Sprague-Dawley machos adultos (com peso corporal de 200 a 220 g) receberam 50 mg/kg de MCT e nenhuma alteração na pressão ventricular direita foi observada em 14 dias. ^32^ Esse resultado divergente foi recentemente elucidado por um estudo em que os pesquisadores examinaram o desenvolvimento de HP-MCT em ratos machos jovens da linhagem Sprague-Dawley (com 4 semanas de idade, pesando cerca de 80 g antes da administração de MCT) e em animais mais velhos (com 17 semanas de idade, pesando aproximadamente 420 g antes da administração de MCT). Os animais mais jovens morreram 23,4 ± 1,1 dias após a administração do MCT, enquanto os animais mais velhos não morreram dentro do período do estudo (42 dias). ^18^ Além disso, no estudo anterior, os autores observaram arritmias no AE (por exemplo, fibrilação atrial) em ratos tratados com MCT; ainda assim, foram utilizados animais mais velhos (200 a 275 g) em comparação com o nosso estudo (~100 g). ^5^ Além disso, os autores administraram 60 mg/kg de MCT, enquanto neste estudo utilizamos uma dose de 50 mg/kg. ^5^

Adicionalmente, é crucial destacar que, no tecido saudável, a propagação da despolarização teve início na área de entrada da veia cava superior no AD, na região do nó sinoatrial, e percorreu o subepicárdio atrial até alcançar a face dorsal do AE, o último a ser estimulado. Contudo, no tecido afetado dos animais com HP-MCT, ocorre a geração quase simultânea de duas ondas de despolarização: uma próxima à entrada da veia cava e outra na área das junções das veias pulmonares no AE. ^12^

Nossos achados demonstram que o AE na HP-MCT pode apresentar uma fisiopatologia distinta do AD, que é mais suscetível ao desenvolvimento de arritmias induzidas pelo mesmo protocolo de estimulação intermitente ( *burst* ). ^22 , 23^

É importante observar que nosso estudo tem algumas limitações: 1) não medimos a pressão média da artéria pulmonar nos ratos; 2) não realizamos o atriograma nos experimentos do AE isolado; 3) estimulamos o AE a uma frequência distante da frequência fisiológica encontrada *in vivo* em ratos.

## Conclusão

Coletivamente, nossos resultados mostraram que há remodelação morfofuncional do AE 20 dias após a administração de MCT em ratos, conforme indicado na Figura Central , o que não aumentou a suscetibilidade do tecido para o desenvolvimento de arritmias *ex vivo* após aplicação do protocolo de estimulação intermitente ( *burst* ).
