# Errata

**DOI:** 10.36660/abc.20230739

**Published:** 2023-11-13

Arq Bras Cardiol. 2023;120(10):e20230188

No Artigo Original “Estudo Morfofuncional do Átrio Esquerdo Isolado de um Modelo Experimental de Hipertensão Pulmonar em Ratos”, com número de DOI: https://doi.org/10.36660/abc.20230188, publicado no periódico Arquivos Brasileiros de Cardiologia, Arq Bras Cardiol. 2023; 120(10):e20230188, na página 1, alterar o nome da autora Fabiana Silva Machado para: Fabiana da Silva Alcântara.

