# Supplementary material for: Estudo Morfofuncional do Átrio Esquerdo Isolado de um Modelo Experimental de Hipertensão Pulmonar em Ratos
Source: Arq Bras Cardiol. 2023 Oct 2;120(10):e20230188. [Article in Portuguese] doi: 10.36660/abc.20230188 (PMC10548886; doi:10.36660/abc.20230188)
Supplement: Translation

# Translation: Isolated Left Atrium Morphofunctional Study of an Experimental Pulmonary Hypertension Model in Rats

**Authors:** Jorge Lucas Teixeira-Fonseca, Julliane Vasconcelos Joviano-Santos, Fabiana da Silva Alcântara, Polyana Leal da Silva, Michael Ramon Lima, Danilo Roman-Campos

**Affiliations:** 1 Brazil Universidade Federal de São Paulo, São Paulo, SP – Brazil; 2 Brazil Faculdade de Ciências Médicas de Minas Gerais, Belo Horizonte, MG – Brazil

**Keywords:** Heart Atria, Fibrosis, Pulmonary Hypertension, Cardiac Arrhythmias, Monocrotaline

## Abstract

**Background:**

The high incidence of atrial arrhythmias in pulmonary hypertension (PH) might be associated with poor prognosis, and the left atrium (LA) may play a role in this. An important finding in PH studies is that LA remodeling is underestimated.

**Objective:**

This study investigated LA morphology and mechanical function, as well as the susceptibility to develop arrhythmias in a monocrotaline-induced PH (MCT-PH) model.

**Methods:**

Wistar rats aged 4 weeks received 50 mg/kg of MCT. Electrocardiography and histology analysis were performed to evaluate the establishment of the MCT-PH model. The tissue was mounted in an isolated organ bath to characterize the LA mechanical function

**Results:**

Compared with the control group (CTRL), the MCT-PH model presented LA hypertrophy and changes in cardiac electrical activity, as evidenced by increased P wave duration, PR and QT interval in MCT-PH rats. In LA isolated from MCT-PH rats, no alteration in inotropism was observed; however, the time to peak contraction was delayed in the experimental MCT-PH group. Finally, there was no difference in arrhythmia susceptibility of LA from MCT-PH animals after the burst pacing protocol.

**Conclusion:**

The morphofunctional remodeling of the LA did not lead to increased susceptibility to ex vivo arrhythmia after application of the burst pacing protocol.

## Introduction

Pulmonary hypertension (PH) is an umbrella term that refers to an increase in mean pulmonary artery pressure (mPAP) ≥ 20 mmHg at rest as assessed by right heart catheterization. ^1^ Lesions in the small pulmonary arteries, including, among others, abnormal proliferation of smooth muscle and endothelial cells in the pulmonary vasculature, ^2^ consequently promotes progressive thickening of the pulmonary artery wall, which results in increased mPAP and gradually induces pressure overload in the cardiac chambers. ^1^

A primary characteristic of PH is the involvement of the right side of the heart. However, as the disease progresses, the left side of the heart, including the left atrium (LA), ^3^ is also affected. ^4 , 5^ The changes in the LA are complex and multifactorial, with an increased area, impaired contractility, and impaired systolic and diastolic function. ^6 - 8^

Chronic stretching of the LA is partly responsible for the supraventricular arrhythmias present in patients with PH. ^3 , 8 , 9^ Possibly, the roof of the LA, characterized by the bifurcation of the pulmonary arteries and the left bronchus, ^10^ is primarily responsible in the genesis of the arrhythmias. ^11^ Several studies suggest that the structural, molecular and functional signature of the right atrium (RA) and LA may be different in PH. ^5 , 12 - 14^

Supraventricular arrhythmias, as seen in PH, are usually associated with disease severity and are related to poor prognosis. ^15^ Thus, understanding the LA remodeling and its susceptibility to develop arrhythmias is of utmost relevance. There are different preclinical models for the study of heart diseases secondary to PH; among these, the monocrotaline (MCT) animal model is undoubtedly the most widely used. MCT is a pyrrolizidine alkaloid extracted from the stems, leaves, and seeds of Crotalaria spectabilis. In rats, MCT is biotransformed in the liver into its active metabolite. The lesions promoted by MCT are heterogeneous, including endothelial injury, medial thickening of large arteries, and cardiac hypertrophy. ^4, 5 , 16 - 21^

A previous report ^5^ described impaired LA electrical function in the MCT-PH model; however, it is unknown whether MCT-PH impairs the LA mechanical function and/or increases susceptibility to mechanical arrhythmias. Thus, this study investigates the LA mechanical function 20 days after MCT administration for control (CTRL) and MCT animals.

## Methods

### Animals

All animal handling procedures were approved by the Ethics Committee for Animal Use of the Federal University of São Paulo (number 9073161118). The rats were obtained from the Center for the Development of Experimental Models for Biology and Medicine and housed in institutional animal care facilities on a 12-hour light/dark cycle with food and water available *ad libitum* .

### Experimental Design for Monocrotaline-Induced Pulmonary Hypertension

A simple draw method was used to randomly divide four-week-old male Wistar rats weighing approximately 100 g into two groups: the control group (CTRL) and the experimental group (MCT). After the draw, the animals were regrouped and identified in their respective groups: 1) CTRL, which received a single i.p. dose of dimethyl sulfoxide (DMSO, Synth®) vehicle solution (1 ml/kg); and 2) MCT group, which received a single i.p. dose of MCT (50 mg/kg - SIGMA Chemical Co., St. Louis, MO, EUA) dissolved in DMSO on day 0, as previously described. ^22 , 23^ The animals were observed for 20 days after MCT and DMSO administration.

### *In Vivo* Electrocardiographic Measurements

The rats were anesthetized by inhalation of 1.5–2.0% isoflurane (Isoforine®, Cristália, SP, Brazil) and placed in a supine position for the electrocardiogram (ECG) experiments. A five-minute ECG recording was performed using electrocardiograph equipment (ECG-PC version 2.07 ®-TEB, MG, Brazil) before the administration of MCT or vehicle and after 20 days of treatment. The parameters evaluated were P wave duration, QRS complex duration, PR intervals, QT interval, and heart rate. The QT interval values were corrected using the Bazett formula (QTc = QT/√RR). All ECG tracings were analyzed offline. At least ten successive electrical cycles were averaged for each animal, and the parameters were analyzed.

### Morphological Parameters

The body weight of the CTRL and MCT rats was measured on day 0 and day 20. At the end of the study, the rats were decapitated for heart collection. We used Fulton’s index to express the degree of right ventricle (RV) hypertrophy, calculated from the weight ratio of the RV to the sum of the left ventricle (LV) and septum (S), as previously described. ^23 , 24^ In addition, the ratio of heart weight to tibia length was evaluated to determine overall heart hypertrophy.

### Histological Studies

The histological processing of the hearts was performed as previously described. ^25^ Tissues were fixed by immersion in 4% paraformaldehyde for 24 hours at 4 °C. Subsequently, the cardiac tissue was dehydrated using an increasing series of ethanol (70, 80, 90 and 100%), followed by clearing in xylol and embedding in paraffin. Then, 8 µm-thick cross-sections were cut using a microtome (model HM335E; Microm, Inc., Minneapolis, MN, USA). After deparaffinization with xylene and rehydration through graded concentrations of ethanol (100, 90, 80, and 70%), the slides were stained with Masson’s Trichrome to assess cardiac morphology, as well as the extent of fibrosis. Grading morphometric analysis was performed using ImageJ ® software, version 1.44 (National Institutes of Health, Bethesda, MD, USA). In this analysis, we used a predefined grid and a cell counter tool for measuring, set with 1,000 grid intersection points per rat from 20 frames, as previously described. ^25^

### Left atrium preparation

The LA was cut perpendicularly and mounted in a tank for isolated organs containing Tyrode’s solution (mM): 140 NaCl, 5.4 KCl, 1.8 CaCl _2_ , 0.5 MgCl _2_ , 0.33 NaH _2_ PO _4_ , 11 Dextrose and 5 HEPES continuously gasified with 100% O _2_ as previously described. ^22 , 23 , 26^ The ends of the LA were suspended horizontally by stainless steel hooks and equilibrated under a resting tension of 0.5 gf (4.9 mN) for at least 40 minutes before recording. The LA was paced at 1 Hz.

### Pacing Protocols

The susceptibility of the LA to develop arrhythmia was tested by applying a single train of 50 pulses at a frequency of 30 Hz through a pair of silver electrodes (Ag/AgCl) positioned at 90º and connected to a stimulator (Myopacer, IonOptix), as previously described. ^27^

### Statistical Analysis

All data are presented as the mean ± standard deviation (SD). The normality of the dependent variable was tested with the Shapiro-Wilk test. Comparisons between groups were performed using a two-tailed unpaired Student’s t-test. Continuous variables were analyzed using the paired Student’s t-test. Fisher’s exact test was used for categorical data (incidence of arrhythmias). Each figure legend identifies the statistical test. The level of significance to reject the null hypothesis was p < 0.05. The number of animals, indicated in the figure legend, is represented by (n). Data were analyzed using Excel ® (Microsoft, USA) and Origin 8.0® (OriginLab, USA).

## Results

### Morphofunctional Analysis of the Heart in the Experimental Model of MCT-PH in Rats

First, we performed a morphofunctional analysis of the heart to observe classical markers of heart disease in the MCT-PH rat model ( Figure 1 ). Initially, we evaluated the heart weight (Figure 1A), the normalized heart weight by body weight (Figure 1B), the right ventricle (RV) weight normalized by tibia length (Figure 1C), and the Fulton’s index (Figure 1D). All these parameters were increased in the MCT group compared to the CTRL group, indicating remodeling of the right side of the heart, as already shown in previous studies. ^5 , 22 , 23 , 28^ Additionally, we found that the LA (Figure 1E) and the LV (Figure 1F) weights were also increased in the MCT group compared to the CTRL group, suggesting structural remodeling of the left side of the heart. In order to further characterize the MCT model, we performed a histological evaluation of the left side of the heart ( Figure 2 ).

**Figure 1:** – Morphological remodeling of the heart after MCT administration. (A) Heart weight; (B) Ratio of normalized heart weight to body weight; (C) Right ventricle (RV) weight normalized to tibial bone length; (D) Fulton’s index; (E) Left atrium (LA) weight; (F) Left ventricle (LV) weight (n=7 animals per group). Data are expressed as mean ± SD. Differences between groups were analyzed using Student’s t-test. * comparing MCT to the CTRL (p < 0.05).

**Figure 2:** – Structural remodeling of the heart after MCT administration. Representative images of transverse sections of cardiomyocytes stained with Masson’s Trichrome (scale bar = 500 µm) from the left atrium (LA) and left ventricle (LV) of control (CTRL, A) and monocrotaline (MCT, B) treated animals; (C) Analysis of mean cross-sectional area (CSA) of the left atrium and ventricle cardiomyocytes; (D) Quantification of collagen deposition. Interstitial fibrotic areas are stained in blue; (E) Cardiomyocyte occupation. (n=3-4 animals per group). Data are expressed as mean ± SD. Differences between groups were analyzed using Student’s t-test. * comparing MCT to the CTRL (p<0.05).

The histological evaluation showed that the cross-sectional area of LA and LV myocytes in the experimental group was enhanced compared to the CTRL group (Figures 2A-B) and quantified in (Figure 2C). Interestingly, this increase in the cross-sectional area was observed in a scenario of increased collagen content in both LA and LV (Figure 2D) and reduced total myocyte occupancy (Figure 2E).

Thus, the MCT-PH model recapitulates most of the morphological heart phenotype observed in other studies. ^5 , 22 , 23 , 28^ In order to confirm that, we explored if the MCT-PH model in our study presents the same expected electrocardiogram changes previously reported in this animal model. ^5 , 22 , 23 , 28^ The surface electrocardiogram was conducted in the control and experimental groups, which is summarized in ( Figure 3 ). Representative ECG traces are shown in (Figure 3A). After 20 days of MCT administration, P wave duration (Figure 3B), PR interval (Figure 3C), and QT interval (Figure 3E) were increased. No changes in the QRS interval were observed (Figure 3D). Thus, the ECG associated with heart morphometric and histological analysis demonstrates that the MCT-PH rat model develops a heart phenotype associated with impaired structure and function, as previously described. ^5 , 22 , 23 , 28^

**Figure 3:** – Electrocardiographic parameters are altered after MCT administration. A) Representative traces; B) P wave duration; C) PR interval; D) QRS complex duration; E) QT interval duration. Data are expressed as mean ± SD (n=7 animals per group). Differences between groups were analyzed using paired Student’s t-test. * comparing to the CTRL (p<0.05).

### LA Function in the CTRL and MCT Rats

Next, we conducted a series of experiments to study the function of *ex vivo* LA paced at 1 Hz. Figure 4 summarizes our findings. A superimposed averaged normalized curve is shown in (Figure 4A) for the CTRL and MCT experimental groups. There was no difference in the LA from the MCT-PH regarding the pacing frequency (Figure 4B) and peak amplitude (Figure 4C). A modest but significant difference was observed in the time to peak contraction (Figure 4D), measured as the time between 10 and 90% for the peak of contraction. Non-change in the time for relaxation was observed (Figure 4E).

**Figure 4:** – The MCT impairs LA function. (A) Representative tracings of normalized superimposed left atrium paced at 1 Hz for control (CTRL) (n=10) and MCT (n=10); (B) Left atrium frequency; (C) Peak amplitude of contraction; (D) Time to peak contraction rise between 10-90%; (E) Time to peak contraction decay between 10-90%. Data are expressed as mean ± SD. Differences between groups were analyzed using Student’s t-test. * comparing to the CTRL (p<0.05).

### Susceptibility to Arrhythmia of Isolated LA From MCT Rats

A previous report found that the isolated RA from MCT-PH animals, after 14 days of MCT administration, is susceptible to developing arrhythmias after *ex vivo* burst pacing protocol. ^22 , 23^ Here, we explored the susceptibility of LA from MCT-PH animals to develop arrhythmias after 20 days of MCT administration. The summary of our findings is found in ( Figure 5 ). After the burst pacing protocol, the CTRL LA developed some extrasystoles as observed in the representative trace (Figure 5A), left trace, and the LA from MCT-PH, represented in (Figure 5A), right trace. As is shown in (Figure 5B), arrhythmic events were observed at the same frequency in the MCT (3/10) compared to the CTRL (5/10) group after burst pacing. Also, most arrhythmic events observed were extrasystoles, and non-atrial fibrillation was detected.

**Figure 5:** – Susceptibility to arrhythmia in isolated LA A) Representative tracings of the isolated LA in the CTRL (left panel) and MCT (right panel) situation after the intermittent stimulation protocol (burst). (B) Incidence of arrhythmic events. Chi-square test, p>0.05. CTRL (n = 10) and MCT (n=10).

## Discussion

The MCT-PH model in rats induces complex cellular and molecular level changes, which contribute to myocardial remodeling, resulting in increased pressure in the cardiac chambers and hypertrophy. In our study, the MCT-PH model was established by an increase in mean LA weight, LV, Fulton index and cardiomyocyte cross-sectional area. ^22 , 23^ In addition, the histological findings confirmed the presence of tissue fibrosis; this phenomenon reflects greater electrical heterogeneity, compromising the atrial structure and, consequently, increasing vulnerability to arrhythmias. ^28^

In the present study, we explored the morphofunctional changes of the LA after MCT administration in rats. However, we found that the MCT-PH model did not increase the susceptibility of the LA to develop arrhythmia *ex vivo* after the burst pacing protocol. Our results differ from the previous report, which used a similar experimental model as the one applied in this study, whose authors found that the LA was susceptible to developing arrhythmias after *in situ* administration of a burst protocol. ^5^ They identified that the susceptibility to arrhythmia was increased in the LA in a scenario of enhanced fibrosis in the tissue and increased P wave duration in a similar fashion as we observed in our current investigation. ^5^

There are some explanations for the apparent discrepancy from our study and that reported by Hiram et al. For example, they applied the drug Blebbistatin in their preparation, which uncouples the electrical and mechanical function of the myocyte. Therefore, their study’s experimental concept differs from ours since we explored the mechanical function to evaluate susceptibility to arrhythmia. They also applied a distinct protocol to induce arrhythmia, which was more severe than ours. They used a 50-Hz burst pacing applied for 3 s, with 12 bursts separated by 2-s intervals; meanwhile, we applied a single 30 Hz burst pacing stimulus.

Another possible explanation for the difference between our results and those in the literature is the age of the rats used in the studies, which will be further discussed. Also, we may say that the time course of MCT-PH disease from our study and others are different, especially related to the dose of MCT and the weight/age of the animals used. ^5 , 22 , 23 , 28^ Here, we report the phenotype of the LA from animals weighing 100 g at the beginning of the study and treated with 50 mg/kg MCT, which is much younger than that used in the study by Hiram et al.

It is already known that animals with ~100 g are more susceptible to MCT administration, with 100% mortality up to 23 days post-MCT treatment. ^18 , 29 , 30^ An interesting observation is that, in another report, it was noted that male Wistar rats weighing 150 to 175 g and administrated with 40 or 60 mg/kg of MCT, after 14 days, both groups developed elevated right ventricular pressure, being more severe in the group that was given 60 mg/kg of MCT. ^31^ Besides, adult male Sprague-Dawley rats (body weights of 200 – 220 g) were given 50 mg/kg of MCT and no change in the right ventricular pressure was observed at 14 days. ^32^ This divergent result was recently explained by a study in which authors evaluated the development of MCT-PH in young male Sprague-Dawley rats (4-week-old, weighing ~80 g before MCT administration) and older animals (17-week-old, weighing ~420 g before MCT administration). Younger animals died 23.4 ± 1.1 days after MCT administration, while older animals did not die within the study’s time frame (42 days). ^18^ In addition, in the previous study, the authors observed arrhythmias in the LA (e.g., atrial fibrillation) in MCT-treated rats; still, older animals (200 to 275 g) compared to our study (~100 g) were used. ^5^ Also, they administered 60 mg/Kg of MCT, ^5^ while in this study we used 50 mg/kg of MCT.

Furthermore, it is important to stress that, in the healthy tissue, the depolarization wave started in the region of the inflow of the superior vena cava into the RA, which is the region of the sinoatrial node, along the atrial subepicardium to the dorsal side of the LA, which is the last excited. However, in the diseased tissue from MCT-PH animals, almost two concomitant depolarization waves are formed: one near the inflow of the vena cava and another in the region of the lacunae of the pulmonary veins in the LA. ^12^

Our findings demonstrate that the LA in MCT-PH may present a distinct pathophysiology from the RA, which is more susceptible to developing arrhythmias induced by the same burst pacing protocol. ^22 , 23^

It is important to note that our study has some limitations: 1) we did not measure the mean pulmonary artery pressure in the rats; 2) we did not perform the atriogram in the isolated LA experiments; 3) we paced the LA in a frequency far from the physiological frequency found *in vivo* for rats.

## Conclusion

Collectively, our results showed that there is LA morphofunctional remodeling 20 days after administration of MCT in rats, as indicated in the Central Illustration , which did not increase the susceptibility of the tissue to develop *ex vivo* arrhythmias after a protocol of burst pacing.
