# Supplementary material for: Errata
Source: Arq Bras Cardiol. 2023 Nov 13;120(11):e20230739. [Article in Portuguese] doi: 10.36660/abc.20230739 (PMC10697678; doi:10.36660/abc.20230739)
Supplement: Translation

# Translation: Erratum

Arq Bras Cardiol. 2023;120(10):e20230188

In the Original Article “Isolated Left Atrium Morphofunctional Study of an Experimental Pulmonary Hypertension Model in Rats”, with DOI: https://doi.org/10.36660/abc.20230188, published in the journal Arquivos Brasileiros de Cardiologia, Arq Bras Cardiol. 2023; 120(10):e20230188, on page 1, change the name of the author Fabiana Silva Machado to: Fabiana da Silva Alcântara.
